# Alterations in exocrine pancreatic function after acute pancreatitis

**DOI:** 10.1016/j.pan.2024.03.003

**Published:** 2024-03-07

**Authors:** Joseph Bejjani, Mitchell L. Ramsey, Peter J. Lee, Anna Evans Phillips, Vikesh K. Singh, Dhiraj Yadav, Georgios I. Papachristou, Phil A. Hart

**Affiliations:** a Division of Gastroenterology, Hepatology, and Nutrition, The Ohio State University Wexner Medical Center, Columbus, OH, USA; b Division of Gastroenterology, University of Pittsburgh Medical Center, Pittsburgh, PA, USA; c Division of Gastroenterology, Johns Hopkins Medical Institutions, Baltimore, MD, USA

**Keywords:** Exocrine pancreatic insufficiency, Exocrine pancreatic dysfunction, Pancreatic enzymes

## Abstract

Exocrine pancreatic dysfunction (EPD) is a malabsorptive complication of pancreatic disorders that can lead to a host of symptoms ranging from flatulence to diarrhea and contribute to weight loss and metabolic bone disease. It is increasingly recognized to occur after acute pancreatitis (AP), including episodes with mild severity. The risk of developing EPD after AP is influenced by a range of factors, including the degree of acinar cell destruction and inflammation during AP, and persistent structural derangements following AP. In this article, we discuss the epidemiology, pathophysiology, and clinical management of EPD after AP while highlighting key knowledge gaps.

## 1. Introduction

Acute pancreatitis (AP) is the most common disease of the exocrine pancreas, resulting in approximately 300,000 hospitalizations per year in the United States alone [1]. While most patients have a brief clinical course with resolution of acute symptoms, intermediate and long-term sequelae are increasingly recognized. Among these complications, patients with AP are at increased risk for both endocrine and exocrine pancreatic dysfunction during the initial hospitalization and follow up.

Digestive capacity is described as the collective effect of pancreatic and extra-pancreatic digestion and nutrient absorption [2]. Due to compensatory increases in extra-pancreatic lipolysis and reserve of the pancreas, a decrease in pancreatic exocrine function often occurs without a decline in digestive capacity [3–7]. EPI is a malabsorptive complication that develops when the endogenous pancreatic enzyme production, secretion, and/or mixing are altered to the degree that fat digestion is impaired. This manifests with symptoms including flatulence, bloating, and diarrhea, and in advanced cases, steatorrhea and weight loss [8]. Other complications resulting from EPI can include micronutrient deficiencies and metabolic bone disease [9,10].

EPI can develop in most diseases of the exocrine pancreas, but has the highest reported prevalence in chronic pancreatitis (CP), pancreatic cancer, and cystic fibrosis [11]. However, there is growing recognition of the development of EPI following AP [12]. While EPI in the setting of severe and moderately severe AP has been well established, the development in the setting of mild AP remains less characterized. In a recent meta-analysis, it was reported that approximately one-quarter of all AP patients will develop EPI during follow up [8].

In this article, we aim to provide an overview of the epidemiology, summary of the pathophysiology, description of the clinical management, and a highlight of key knowledge gaps for future studies to advance the understanding of EPI following AP.

### 1.1. Search strategy and definitions

We searched for potential references for this narrative review using PubMed with the following items in both Mesh terms and plain text: ((exocrine) AND ((pancreas) OR (pancreatic)) AND ((insufficienc*) OR (dysfunct*))) AND ((“pancreatitis”) AND “acute”)). Additional articles were identified from the reference lists of selected articles. Articles were restricted to those in English language and searched all time through July 1, 2023.

A recent staging system has been proposed to distinguish exocrine pancreatic insufficiency (EPI) from a broader concept termed exocrine pancreatic dysfunction (EPD) [1]. EPD also encompasses the situation where there is a deficit in production, secretion, and/or pancreatic enzyme mixing, but not to the degree that fat malabsorption is present. The staging system proposed by Khan et al. considers symptoms, fecal elastase (FE-1) measurements, coefficient of fat absorption (CFA), and fat-soluble vitamin levels [2]. Most studies were published prior to this proposed system with EPI and EPD used interchangeably. We are unable to retrospectively harmonize these data, so we have elected to use the broader term of EPD to collectively refer to both EPI and EPD. Lastly, studies have used different thresholds for classification of exocrine function when assessed using FE-1. The most common FE-1 thresholds for identifying EPD were <200 and < 100 mcg/gm.

## 2. Epidemiology

The incidence of AP is increasing globally, with higher rates observed in North America compared to other regions [13]. In the United States, AP is responsible for greater than 300,000 hospitalizations per year, with healthcare expenses of more than $2 billion USD annually [1,14].

Due to the low mortality rate, attention is shifting to better understand and ultimately prevent the intermediate and long-term complications [13,15].

EPD is increasingly recognized as a sequalae of AP. Recent meta-analyses reported a point prevalence of EPD of 62% during hospitalization, and persistent EPD during long-term follow up for 25–35% of patients [8,16]. Of those with EPD, there was a slightly higher proportion with mild severity (defined as FE-1 of 100–200 mcg/gm) compared to severe severity (defined as FE-1 <100 mcg/gm) [16]. The natural course of EPD in the context of AP is not clearly defined, particularly in regards to its occurrence during or following an episode of AP, and whether EPD persists or resolves over time [8,16].

Multiple factors have been identified that influence the incidence of EPD after AP, including AP etiology, AP severity, and the presence of pancreatic necrosis. One meta-analysis found that patients with alcohol-related AP were nearly twice as likely to develop EPD during follow up as those with non-alcohol etiology; this question has not been well studied in the setting of chronic pancreatitis. Additionally, patients with organ failure and/or necrotizing AP were 50% more likely to develop EPD compared to patients with mild or interstitial AP [8]. Though considered to be at lower risk of developing EPD after AP, patients with mild AP can still develop EPD at moderately high rates of almost 50% during admission and 20% during follow up [16].

It is important to acknowledge that prevalence estimates from recent meta-analyses reported a high statistical heterogeneity, which could not be explained by sensitivity or subgroup analyses [8,16]. In addition to the pancreatitis-related characteristics, there are multiple aspects of study design that could potentially influence prevalence estimates, including eligibility criteria, duration of follow-up, and method of assessing exocrine function. Regarding the last consideration, it is important to note that the prevalence of EPD is significantly higher (42% vs. 24%) when using direct vs. indirect pancreatic function testing [8]. Moreover, estimates differ when comparing different methods of indirect pancreas function testing (namely coefficient of fat absorption and FE-1); however, the reported differences are paradoxical and may merely reflect differences in study populations or other differences in study design [16].

## 3. Pathophysiology

The changes in exocrine function during and following an AP episode are poorly characterized, which is largely due to the lack of direct, empirical data in humans. Precisely estimating the prevalence of EPI after AP is challenging due to a variety of definitions in the literature and lack of consensus on the optimal diagnostic tool. Similarly, the mechanisms underlying the development of EPI after AP are incompletely described, but can be due to decreased pancreatic enzyme production and/or impaired secretion [17]. In addition to the usual challenges with studying exocrine function, the use of direct pancreatic function testing is generally not advised due to the theoretical concern of exacerbating pancreatic injury by providing exogenous hormonal stimulation. With that disclaimer, there are multiple hypothesized defects involving various pancreatic structures, which we elaborate (Table 1). Based on the evolution of AP from an immunological, symptomatic, and imaging standpoint, it is expected that the relative contribution of different factors varies at different stages of AP evolution. Additionally, non-pancreatic factors may contribute to EPD, such as small bowel mucosal dysfunction causing disordered prandial hormone responses and ultimately resulting in decreased pancreatic secretion [17].

### 3.1. Acinar cell

The pancreatic acinar cells produce and secrete digestive enzymes into the small intestine [18]. During AP, acinar cells are damaged and may temporarily reduce protein synthesis or undergo apoptosis, resulting in a reduction in functional acinar cell mass [19,20]. Much has been written on the acinar cell response to stress, and many of the described mechanisms result in decreased enzyme production which has a direct impact on the development of clinical EPD. In this section, we will first discuss possible mechanisms for the clinical observation of “stunned” exocrine function followed by potential mechanisms responsible for permanent loss of exocrine function.

Cellular stresses within acinar cells lead to alterations in protein synthesis, trafficking, and secretion, resulting in vacuole accumulation and alterations in autophagy [21]. Together, these cellular responses temporarily diminish enzyme production. However, these cellular responses may avoid apoptosis, so pancreatic enzyme secretion can be restored once cellular homeostasis is regained. Avoidance of apoptosis by suspending enzyme production may partly explain the observation that many patients will experience some degree of EPD in the first month after AP followed by improvement [22]. As inflammation resolves, many individuals will recover pancreatic function; those with persistent EPD may reflect either a more severe injury to acinar cells leading to apoptosis or an alternative mechanism of injury [23].

When acinar cells are stressed beyond limits of homeostasis, apoptosis occurs. Apoptosis leads to the release of pro-inflammatory cytokines and chemokines which results in the recruitment of immune cells to the acinar cells [24–26]. Recruitment and activation of immune cells at the level of the acinus further contributes to autodigestion of the pancreas, resulting in a permanent loss of exocrine cell mass and predisposing to the development of EPD [27,28]. During the initial phase of AP, when acinar cell stress is most severe, the prevalence of EPD may exceed 80% in patients with severe AP or pancreatic necrosis [29]. Even though the inflammatory component wanes over time, persistent EPD could be attributed to loss of acinar cell mass due to apoptosis. Additional mechanisms, including ductal changes, can also contribute to permanent EPD.

### 3.2. Ductal cell

During AP, a reduction in secretin release is expected, ultimately leading to a reduction in the neutralization of chyme in the duodenal lumen due to decreased bicarbonate secretion [30,31]. In addition to changes in cellular function, AP can involve gross disruption of the pancreatic duct leading to additional pancreatic injury and secondarily to malabsorption and malnutrition [17]. In the recovery phase, it has been proposed that fibrosis can produce pancreatic duct strictures, similarly reducing the flow of pancreatic fluid [17].

### 3.3. Islet-acinar axis

The relationship between the islets and acinar cells of the pancreas, known as the “islet-acinar axis”, plays a crucial role in maintaining endocrine-exocrine function [32]. There is a close relationship between diabetes mellitus and exocrine function, as diabetes can contribute to the development of EPD and long-standing EPD has been associated with diabetes [33,34]. There are several potential causes of EPD in individuals with diabetes, including the absence of insulin’s stimulatory effects on acinar cells, autoimmunity leading to destruction of both endocrine and exocrine tissue, and decreased pancreatic exocrine secretion as a result of diabetic neuropathy [35,36]. We speculate that the disruption of these homeostatic relationships as well as more widespread impairment of hormonal mediators and/or neural stimuli further contribute to changes in exocrine function [37].

### 3.4. Stellate cells

Pancreatic stellate cells play a crucial role in maintaining the pancreatic extracellular matrix. Following pancreatic injury, stellate cells are activated and expand the extracellular matrix, which is eventually replaced with fibrosis [38]. It isn’t clear if a single episode of AP is sufficient to produce fibrosis severe enough to produce clinical sequelae; however, an expanded and fibrotic stroma could contribute to the development of EPD by causing pancreatic ductal strictures or replacement of acinar cells, similar to chronic pancreatitis [38].

Beyond fibrosis, stellate cells have been purported to behave as immune cells by engulfing damaged acinar cells and as mediators of cholecystokinin-induced acinar cell secretion [38]. In the context of AP, stellate cells may contribute to long term EPD by reducing the number of functional acinar cells, or may contribute to transient EPD by temporarily limiting the effects of cholecystokinin on enzyme secretion [39,40]. Activated stellate cells also propagate local inflammation by secreting pro-inflammatory cytokines [38]. Stellate cells may be deactivated through apoptosis, return to quiescence mediated by vitamin A, or through cellular senescence [41]. While the exact mechanism has not been elucidated in humans, deactivation of stellate cells likely has implications for the long term development of EPD and remains a topic of interest for future study.

### 3.5. Other considerations

In addition to influencing the risk for developing AP, genetic factors can contribute to the development of EPD. The most direct example is that patients with pathogenic variants (PV) in the *cystic fibrosis transmembrane conductance regulator gene (CFTR)* are at increased risk for developing EPD. In fact, EPD can even develop *in utero* for individuals with two severe *CFTR* PVs [42]. The CFTR protein is expressed on pancreatic ductal cells and loss-of-function PVs lead to alterations in ductal ion transport channels leading to increased viscosity of the pancreatic fluid and reduction in the alkalization of the duodenal chyme, both contributing to EPD [43]. The relationship between CFTR function and EPD has been strengthened by recent studies which have shown that CFTR modulating drugs improve fat absorption in some individuals, presumably due to restoration of ductal secretion [44–46]. While the mechanism is less evident, patients with a PV in the *Serine Protease Inhibitor Kazal-type 1* (*SPINK1)* gene and, to a lesser degree, *Cationic trypsinogen* (*PRSS1*) also have an increased rate of progression towards EPD [47,48].

Environmental factors are likely mediators of exocrine function and recovery following AP, and may partly explain the development of EPD in patients with mild AP. In retrospective series, prolonged and excessive alcohol consumption has been one of the most consistent risk factors for EPD after AP [8,16]. This is not surprising due to prior observations that heavy alcohol consumption contributes to injury to acinar, ductal, and pancreatic stellate cells [37,49]. Additionally, cigarette smoking initiates and/or propagates pro-inflammatory and pro-fibrotic pathways that contribute to reduced exocrine (and endocrine) function [50]. Furthermore, both alcohol consumption and smoking can impair CFTR function further contributing to the previously discussed ductal dysfunction [51,52].

AP may occur in individuals with a structurally abnormal pancreas, such as pancreatic cancer or non-alcoholic fatty pancreas disease (NAFPD), and these individuals have a high risk of developing EPD. While AP could further contribute to risk for EPD for individuals with pancreatic cancer, the prevalence of EPD is already high (approximately 70%) due to other anatomical factors [53,54]. The primary predictors of EPD in pancreatic cancer include tumor location in the pancreatic head, a surgical loss of pancreatic tissue, and the presence of ductal obstruction [31,55,56]. The prevalence of EPD among individuals with NAFPD is less established, and the exact mechanism of EPD in NAFPD is also unknown. This may be mediated through acinar cell destruction or may be related to dysregulated crosstalk between the endocrine and exocrine compartments of the pancreas in the setting of diabetes [57]. Indeed, EPD, pancreatic steatosis, and diabetes are closely related, and AP may occur in these individuals [58–60]. In a recent multicenter, prospective study, the incidence of diabetes was found to be approximately 10% at 1-year following AP [61]. Additionally, many patients with pancreatogenic diabetes exhibit signs of EPD [57]. The incidence of EPD and diabetes after AP may share similar risk factors, and this topic remains an active area of investigation.

Lastly, repeated episodes of AP can impair the pancreas’ ability to recover, potentially leading to permanent loss of digestive enzyme secretion and/or CP. While the exocrine pancreas has the capacity to regenerate in murine models, there has been no direct evidence of regeneration in humans. Thus, while many individuals fully recover from AP, even a single episode can have potentially permanent effects [27,28].

## 4. Management

There is limited information regarding the proper management of EPD after AP. For example, systematic screening for signs and symptoms of EPD is not addressed or recommended in most of the current guidelines for AP. Nevertheless, available epidemiological data and best practices from managing EPD in other disease states can be used to guide medical decision-making regarding prevention, screening, and treatment of EPD after AP.

The risk of developing EPD is closely tied to the severity of AP, so primary prevention is related to measures that mitigate the progression of mild AP. The initial management step in this regard is to initiate early, goal-directed fluid resuscitation, avoiding an aggressive resuscitation approach [62,63]. Additional pharmacological interventions are being studied in the acute phase, and the effects on development of EPD can be considered as a secondary outcome. Patients with modifiable risk factors for the development of AP and disease progression should be encouraged to make lifestyle changes, including abstaining from alcohol and smoking cessation. Importantly, stopping alcohol consumption has been shown to be a key factor in recovery for patients with alcohol-related AP, as only 6% developed EPD during longer term follow-up when abstinent [64].

Assessment for EPD in the acute (hospitalization) phase of AP is challenging where the diagnosis needs to be differentiated from other potential problems directly or indirectly related to AP that can contribute to overlapping symptoms of diarrhea and/or weight loss. While pancreatic function testing would ideally be employed to provide diagnostic clarity, this is rarely used due to limitations with completing the testing and/or limitations in accuracy, particularly during hospitalization [65]. In patients with advanced clinical severity and/or prolonged hospitalization with suboptimal nutritional intake where there is higher likelihood for EPD and/or less physiological reserve to tolerate additional nutritional compromise, it is likely prudent to err on the side of early institution of pancreatic enzyme replacement therapy (PERT).

In the recovery (post-hospitalization) phase it remains important to maintain an index of suspicion for underlying EPD. Screening for EPD after AP is not addressed in most current guidelines for the management of AP, which primarily focus on early treatment. Nevertheless, it is prudent to consider this as a contributor for patients with post-prandial symptoms and/or challenges with weight loss or regaining weight. While this is a key consideration for patients with advanced severity of disease or alcohol etiology, EPD can also develop in the setting of mild AP suggesting it is reasonable to screen all patients with AP. Other authors have recently proposed monitoring FE-1 levels every 6–12 months for up to five years [15]. While this could be a reasonable approach, additional studies to determine the correlation of symptoms and FE-1 levels may clarify if a symptom-based surveillance strategy would be sufficient.

Once EPD is identified, the primary treatment involves administration of PERT [66]. PERT is not routinely administered to AP patients without a diagnosis of EPD. Studies on the effects of empiric PERT initiation in this population are lacking but will likely require accurate, early recognition of high-risk groups to target with empiric treatment. Once started, PERT can be optimized through adjustments to dosage, use of an enteric coating, reduction of gastric acid, and proper timing during meals [67]. Supplementation with fat-soluble vitamins may also be necessary [68]. Generally we recommend starting with a dose of approximately 25,000e50,000 of lipase with each meal (and half the dose with snacks), and titrating to resolution of symptoms. Due to the potential for recovery of exocrine function, the duration of therapy for EPD is not necessarily indefinite in the setting of AP, in contrast to CP or pancreatic ductal adenocarcinoma (PDAC) where loss of exocrine function is typically irreversible. However, approaches for de-escalation of PERT have not been investigated in AP and such studies are needed.

## 5. Knowledge gaps and future directions

While multiple advancements have been made in our understanding of the epidemiology of EPD after AP, there are many remaining knowledge gaps that require systematic investigation. Perhaps the largest barrier to further progress is not unique to AP, but relates to the lack of an accurate and widely available method for assessing exocrine pancreatic function. FE-1 has become the most utilized test in clinical and research environments despite limitations in diagnostic performance, which is likely related to its convenience and lack of alternative options. Other assessment strategies in use or under development include the mixed triglyceride breath test, malabsorption blood test, and erythrocyte fatty acid composition analysis [69–71].

For the current review we have adopted a pragmatic approach to the terminology referring to decreased exocrine pancreatic function as EPD; however, there remains a need for consensus regarding nomenclature. In addition to permitting comparisons across prior studies, this will be important to harmonize outcome assessment in future clinical trials. Khan et al. have recently proposed a classification for distinguishing EPI and EPD [2]. While this approach requires additional validation, it serves as a helpful starting point for discussion. This model, as well as future iterations, needs to be evaluated to demonstrate the ability to discriminate and measure improvements in patient-reported outcomes (including symptoms, quality of life, and/or global sense of well-being) and clinical sequalae (e.g., micronutrient deficiencies) prior to widespread utilization in clinical research or practice. A challenge in this validation work is that there is a paucity of data regarding the correlation between exocrine function (including with surrogates for EPI such as FE-1) and gastrointestinal symptoms or other patient-oriented outcomes. Ongoing observational studies are expected to provide such data, but additional investigations will be needed to understand the role of different dietary patterns (e.g., moderate to low fat intake) as well as the degree of clinical improvement for patients with EPD following dietary and/or pharmacologic interventions [72,73].

Lastly, prospective studies with longitudinal evaluations of exocrine function and assessments of the impact of potential disease- and patient-related factors are underway to identify bio-markers and/or develop prediction scores to improve recognition of EPD after AP [74]. In addition to gaining insights to the potential underlying pathophysiology, these risk factors and diagnostic bio-markers may allow the clinician to provide more tailored screening and avoid both over treatment as well as unnecessary delays in starting treatment.

## 6. Conclusion

EPD is increasingly acknowledged as a complication of AP, resulting in digestive symptoms, contributing to malnutrition, and likely compromising quality of life. While EPD is most prevalent in patients with severe or necrotizing AP, even those with mild AP are at risk of developing EPD. EPD following AP, in contrast to most other exocrine pancreatic diseases may be transient. Screening is expected to improve treatment rates and clinical outcomes, but an optimal screening strategy needs to be developed. Further work is needed to develop a consensus regarding terminology for EPD and a diagnostic test; these fundamental steps will enable additional investigations to better understand the underlying mechanisms and refine the approach to diagnosis and management of patients with EPD after AP.

## Figures and Tables

**Table 1:** Potential factors contributing to changes in exocrine function after acute pancreatitis.

Functional Compartment Contribution to Exocrine Pancreatic Dysfunction After Acute Pancreatitis	
Acinar Cell	
Acinar cell injury	Apoptosis and necrosis of acinar cells reduces functioning acinar cell mass, resulting in diminished pro-enzyme production. Pathogenic variants in PRSS1 and SPINK lead to changes in pro-enzyme production and function predisposing to premature autoactivation and increased injury to acinar cells. Alcohol exposure predisposes to more extensive acinar cell loss during AP.
Disruption of islet-acinar axis	Decreased secretory signaling from damaged islet cells impairs prandial and interdigestive pro-enzyme production.
**Ductal Cell**	
Ductal cell injury	Necrosis of ductal cells may result in disruption or stenosis of the pancreatic duct, leading to impaired flow of enzymes from the acinus to the duodenum. Ongoing inflammation can lead to fibrosis and compression of the pancreatic ducts. Pathogenic variants in CFTR cause reduced duct cell function predisposing to onset of AP. Alcohol and smoking may contribute to reduced ductal cell function before and after AP.
Reduced bicarbonate secretion	Reduction in secretin production and/or response to signaling decreases bicarbonate secretion, which impairs neutralization of chyme and diminishes the efficacy of enzymes (e.g., patients with small bowel dysfunction during/following AP).
**Islet Cell**	
Islet cell injury	Damage to islet cells reduces secretion of stimulatory hormones, including insulin. Loss of insulin production impairs acinar cell function and may lead to diabetes and subsequently diabetic neuropathy, which further impairs the islet-acinar axis
**Stellate Cell**	
Stellate cell activation	Limits cholecystokinin-induced acinar cell secretion. Engulf damaged acinar cells, potentially contributing to long term EPI by reducing functional acinar cell mass. Expands extracellular matrix initially, and eventually leads to ductal fibrosis potentially causing ductal strictures and reduced delivery of enzymes to the duodenum.
**Other**	
Systemic inflammation	Release of pro-inflammatory cytokines and chemokines into pancreatic capillaries results in immune cell recruitment to the pancreas which contributes to injury in all compartments.

## Abbreviations

AP: acute pancreatitis
EPD: exocrine pancreatic dysfunction
EPI: exocrine pancreatic insufficiency
FE-1: fecal elastase-1
PERT: pancreatic enzyme replacement therapy
